# High-Throughput 3D Screening Reveals Differences in Drug Sensitivities between Culture Models of JIMT1 Breast Cancer Cells

**DOI:** 10.1371/journal.pone.0077232

**Published:** 2013-10-23

**Authors:** Vesa Hongisto, Sandra Jernström, Vidal Fey, John-Patrick Mpindi, Kristine Kleivi Sahlberg, Olli Kallioniemi, Merja Perälä

**Affiliations:** 1 Biotechnology for Health and Well-being, VTT Technical Research Centre of Finland, Turku, Finland; 2 Department of Genetics, Institute for Cancer Research, Oslo University Hospital Radiumhospitalet, Oslo, Norway; 3 K.G. Jebsen Centre for Breast Cancer Research, Institute for Clinical Medicine, University of Oslo, Oslo, Norway; 4 Institute for Molecular Medicine Finland (FIMM), University of Helsinki, Helsinki, Finland; 5 Department of Research, Vestre Viken Hospital Trust, Drammen, Norway; Biological Research Centre of the Hungarian Academy of Sciences, Hungary

## Abstract

The traditional method for studying cancer *in vitro* is to grow immortalized cancer cells in two-dimensional monolayers on plastic. However, many cellular features are impaired in these artificial conditions, and large changes in gene expression compared to tumors have been reported. Three-dimensional cell culture models have become increasingly popular and are suggested to be better models than two-dimensional monolayers due to improved cell-to-cell contact and structures that resemble *in vivo* architecture. The aim of this study was to develop a simple high-throughput three-dimensional drug screening method and to compare drug responses in JIMT1 breast cancer cells when grown in two dimensions, in poly(2-hydroxyethyl methacrylate) induced anchorage-independent three-dimensional models, and in Matrigel three-dimensional cell culture models. We screened 102 compounds with multiple concentrations and biological replicates for their effects on cell proliferation. The cells were either treated immediately upon plating, or they were allowed to grow in three-dimensional cultures for 4 days before the drug treatment. Large variations in drug responses were observed between the models indicating that comparisons of culture model-influenced drug sensitivities cannot be made based on the effects of a single drug. However, we show with the 63 most prominent drugs that, in general, JIMT1 cells grown on Matrigel were significantly more sensitive to drugs than cells grown in two-dimensional cultures, while the responses of cells grown in poly(2-hydroxyethyl methacrylate) resembled those of the two-dimensional cultures. Furthermore, comparing the gene expression profiles of the cell culture models to xenograft tumors indicated that cells cultured in Matrigel and as xenografts most closely resembled each other. In this study, we also suggest that three-dimensional cultures can provide a platform for systematic experimentation of larger compound collections in a high-throughput mode and be used as alternatives to traditional two-dimensional screens for better comparability to the *in vivo* state.

## Introduction

The majority of research is carried out using immortalized cells cultured in two dimensions on plastic, but there is growing interest in moving to more *in vivo*-like systems. Three-dimensional (3D) culture systems are starting to claim this spot [1]. A wealth of evidence points to the differences in cells grown in 3D or two-dimensional (2D) culture systems. Cells grown in 2D monolayers or in 3D cultures have been shown to have different protein expression profiles [2]. Differential expression of genes involved in signal transduction [2,3], cellular movement, cell-to-cell signaling, cellular growth, and morphology have also been reported [4,5]. For example, cross-regulation of ß1-integrin and epidermal growth factor receptor (EGFR) pathways occurs in cells cultured in 3D but not in 2D [6]. This was also confirmed *in vivo* using nude mice [7,8]. The cell culture conditions have also been shown to affect human epidermal growth factor receptor 2 (HER2) signaling. HER2 preferentially forms heterodimers in 2D cultures of SKBR3 breast cancer cell whereas 3D culture on poly(2-hydroxyethyl methacrylate) (polyHEMA) plates promotes HER2 homodimerization [9]. The increased homodimerization in 3D leads to increased HER2 activation and its localization to membrane rafts making the cells more sensitive to trastuzumab. The same study also reported that Akt was activated in 2D cultures and downregulated in 3D, whereas MEK1/2 and MAPK levels were increased in 3D. This was also the case for the breast cancer cell lines BT474 and KPL4 [9]. The Pickl et al. study highlights the importance of using 3D culture systems when studying HER2-positive breast cancer as they rely heavily on HER2 and Akt signaling, which are differentially regulated in 3D versus 2D.

Differences in drug responses between 2D and 3D culture models have been reported by several laboratories. However, differences were usually observed using a single drug, which might show increased sensitivity in either 2D or 3D. For example, SKBR3 cells grown in 3D polyHEMA cultures are more responsive to trastuzumab than cells grown in 2D [9], but more resistant to trastuzumab when grown in Matrigel 3D cultures [10]. In addition, Weigelt et al. have shown that the response of the HER2-amplified breast cancer cell lines SKBR3, AU565, and HCC1569 to anti-HER2 agents trastuzumab, pertuzumab, and lapatinib was highly cell line dependent and dependent on whether the cells were cultured on 3D extracellular matrix gels or in 2D monolayers [10]. Phosphorylation of HER2 was significantly reduced in SKBR3 cells cultured in 3D when compared with cells grown in 2D, which could explain the differences in drug responses. Li et al. showed that, in 2D, cell line variants of MCF10 (a normal human epithelial breast cell line) responded similarly to MEK inhibition, whereas in 3D, the carcinoma variant of MCF10 became much more sensitive to MEK inhibitors. This was also observed in MDA-MB-231 (a basal-subtype, breast carcinoma cell line) [11].

In addition to the drug sensitizing effects in 3D, elevated chemoresistance to anticancer drugs in 3D models is also very well characterized [12-15]. Several possible explanations have been suggested for chemoresistance, including increased pro-survival signaling in 3D models, upregulation of genes conferring drug resistance and poor diffusion of drugs. Variations in drug response in xenografts and 3D and 2D cultures have also been studied. For example, in a monolayer culture MGH-U1 (a human bladder carcinoma cell line) cells are more responsive to the cytotoxic effects of Adriamycin (doxorubicin) treatment when compared to cells grown as xenografts or 3D spheroids, most likely due to the poor access of Adriamycin to the internal cell layers of tumors and 3D structures [16].

The purpose of this study was to develop a quick and easy-to-use high-throughput method for drug screening of cells grown in 3D. In parallel with the method development, the biological aspect was to compare the drug sensitivities of the HER2-positive JIMT1 cells grown in 2D and 3D cell culture conditions, and to determine based on gene expression patterns which of the cell culture models mimics *in vivo* tumors most.

## Materials and Methods

### Cell culture

JIMT1 (DSMZ GmbH, Germany) is a HER2+, ER-, PR-, epithelial-like cell line established from the pleural effusion of a 62-year-old woman with trastuzumab-resistant ductal breast cancer [17]. JIMT1 cells were cultivated in a 1:1 mixture of Ham’s F-12 + GlutaMAX medium (Gibco Invitrogen, USA) and Dulbecco’s modified Eagles medium (DMEM, glucose 4.5 g/l; Sigma Aldrich), with 10% fetal bovine serum (FBS), 2 mM L-glutamine, 0.01 mg/ml insulin, and 1% penicillin/streptomycin.850 cells/well were used for the 2D compound screens (2D7d, 7 day culture). DSMZ authenticates all human cell lines by DNA typing using short tandem repeats and additional cytogenetic and immunophenotypic tests. Cells were cultured for a maximum of 30 passages before use.

MCF-7 cells (Interlab Cell Line Collection Italy) were cultivated in Dulbecco’s modified Eagles medium (DMEM, glucose 1 g/l; Sigma Aldrich) supplemented with 10% FBS, 2 mM L-glutamine and 1% penicillin/streptomycin.

### The Matrigel models

In the MG7d (Matrigel 7 days) model, the compound libraries, in 100 nl/well, were pipetted to 384-well plates (Greiner Bio-One, Germany) using an automated liquid handling station (Hamilton Bonaduz AG, Switzerland). 20 µl of ice-cold Matrigel^TM^ dilution (1/3 Matrigel (Basement Membrane Matrix Growth Factor Reduced, BD Biosciences) and 2/3 Opti-MEM® (Life Technologies)) was transferred to drug plates with a Multidrop 384 Microplate Dispenser (Thermo Labsystems, Thermo Electron Corporation, MA) after which the Matrigel was left to polymerize in a cell culture incubator for 20 minutes. The Matrigel amount, or the gel thickness, was optimized so that the cells did not penetrate through the gel and form a 2D culture on the bottom of the wells in the maximum assay time of 11 days. Cells in warm culture medium were added on top of the Matrigel and cultured for the desired time period.

For the MG4+7d (Matrigel 4+7 days) model, the cells were plated on top of the Matrigel as above for a total of 40 µl. At day 4, after the cells had formed 3D structures, the automated liquid handling station was used to pipette the compound library in 10 µl/well in cell culture medium on top of the cultures. 500 cells/well in a total volume of 50 µl/well were used for both Matrigel models.

### The polyHEMA-induced anchorage-independent 3D models

384-well plates were coated with 35 µl/well of PolyHEMA (poly (2-hydroxyethyl methacrylate), Polysciences Inc.), ethanol (>99%) and sterile water in a 4:90:6 ratio. The plates were left to dry for 7 days at 37°C. PolyHEMA creates a synthetic hindrance for the cells and inhibits them from attaching to the plates [18]. Cells thus form 3D structures by attaching to each other. For the PH7d (polyHEMA 7 days) model, the compound libraries were pipetted to the coated plates (10 µl/well), and 40 µl of cell-suspension/well were then added to plates. For the PH4+7d (polyHEMA 4+7 days) model, the cells were allowed to grow and form 3D structures for 4 days in the polyHEMA plate (in 40 µl) before the compounds (10 µl) were added. 5000 cells/well were used for both polyHEMA models.

### Cell viability assay

Cell viability was measured with the CellTiter-Glo (CTG, Promega) luminescent assay using the EnVision Plate Reader (PerkinElmer Inc.) 45 µl of reagent was used for the 3D models, and 25 µl for 2D. This disrupted the 3D and 2D cultures, which was confirmed with microscopic visualization. No background signal was detected from wells containing only Matrigel or polyHEMA and culture medium (data not shown).

### Caspase-3/-7 activity assays

For caspase-3 and -7 activity measurements, JIMT1 cells were plated on 384 well plates and grown in 2D, Matrigel, or polyHEMA for the indicated times. A 1:1 ratio, or 50 µl, of Caspase-Glo 3/7 reagent (Caspase-Glo 3/7 assay, Promega) was added to the wells, and the plate was placed on an orbital shaker for 1hour after which the luminescence was read with the EnVision Multilabel Plate Reader (PerkinElmer).

### Compound screens

The MicroSource cancer compound library consisting of 80 compounds was used (File S1 for annotation). The compounds and controls were plated in four different concentrations using the Hamilton robotic liquid handling station. The final concentration range for the compounds was 0.02, 0.2, 2, and 20 µM per well.

In addition, a 22-drug library consisting mainly of HER2+ cancer-specific drugs was screened using seven concentrations in two technical replicates (as specified in File S1). Both libraries were screened three times.

Drugs that induced more than 30% response (decrease in cell viability) when compared to the controls in any of the five models were selected for further analysis. Of the 102 drugs, 63 fulfilled these criteria. The loess-log normalized screening data was expressed drug-wise as the percentage of the lowest concentration (the two lowest concentrations for the 22-drug screen due to the lower concentration starting point), and the 63 drug average responses from each model were compared. Statistical analysis was done using a t-test. No interference with the 3D sphere formation was observed with any of the compounds.

### Compound screen normalization

Screening data were processed through an automated analysis work-flow implemented in R that takes in raw HTS experiment data files from the plate reader along with bar code-linked, plate-specific annotation files, performs various normalizations, and generates a comprehensive visual output as well as interactive Excel files for initial hit finding.

The loess-log normalization algorithm used for this screen is a novel, statistically more robust, screening data normalization method that down-weights outliers on the plate before calculating the loess fit. After loess correction, the data are normalized to the negative controls and log_2_-transformed. Data aggregation was done by fitting a linear model to loess-log-normalized screening data. The least-squares method is used to estimate the coefficients. After centering to the plate median, hits were determined using the RankProd R package [19] by comparing each 3D condition to the 2D condition and treating each concentration as an individual origin.

### Multiple linear regression (MLR) analysis of the compound screen data

We performed multiple linear regression analysis comparing drug responses in 3D models to the 2D model to study differences in drug responses between the cell culture models used. These differences were explored using the slope coefficients (estimates) from the linear regression model. The estimate shows the rate and direction of the responses compared to those of the control (2D7d). Cell culture models with significant (p-value < 0.05) negative estimates were identified as more sensitive models than the 2D cell culture model.

### Gene expression and xenografts

For the gene expression studies, JIMT1 cells and MCF7 cells were grown in 6 well plates in a 2D (7d), PH (4 and 7 days), or MG (4 and 7 days) model. Matrigel was dissolved before RNA was isolated using Dispase (BD Biosciences). To obtain xenograft tumors, the fat pads of BALB/c-nude mice were injected with 1x10^-6^ JIMT1 cells or MCF7 cells in 25 µl of medium and 25 µl of Matrigel. The tumors were collected 43 days after injection at 99-158 mm^2^. Tumor size was calculated from palpation results using (length/2 x width/2) x π. RNA was isolated using a mirVana (Life Technologies) kit according to the manufacturer’s instructions. Gene expression was analyzed using Illumina HumanHT-12v 4.0 Expression BeadChip (

> 47 000 probes). Two biological replicates were used for each sample. Gene expression results have been deposited in the NCBI Gene Expression Omnibus [20] and are accessible through GEO Series accession number GSE42529 for JIMT1 and GSE47583 for MCF7. Publicly available gene expression datasets were used for MDA-MB-231 and Hs578T cell lines (GEO accession number GSE36953).

Hierarchical clustering of the gene expression data was done using the pvclust R-package using correlation as the distance parameter and averaging as the clustering method [21]. Ingenuity pathway analysis (IPA) and VENNTURE [22] programs were used for the data analysis.

### Gene expression data normalization

The data were analyzed and visualized using standard and custom algorithms implemented in the R/BioConductor framework for statistical computing [23]. Raw data was transformed using the variance-stabilizing transformation method described by Lin et al. [24] and implemented in the lumi package [25]. A linear model was fit to the data and differential gene expression was assessed by computing empirical Bayes statistics as implemented in the limma package [26].

### Validation of gene expression results

The gene expression levels of 11 genes shown in Table 1 and Table 2 were validated with quantitative RT-PCR using TaqMan in the JIMT1 cells. In short, total cellular RNAs were isolated with the RNeasy RNA Isolation kit (Qiagen). Matrigel was dissolved using Dispase (BD Biosciences) before the RNA was isolated according to the manufacturer’s instructions. For cDNA synthesis, 100 ng of total RNA was reverse transcribed with a High Capacity DNA Reverse Transcription kit (Applied Biosystems, Foster City, CA, USA). Thereafter, the cDNAs were diluted 1/10 and TaqMan quantitative real-time-PCR analysis was conducted with an Applied Biosystems 7900HT instrument using specific primers for the amplicon genes and beta-actin designed by the Universal ProbeLibrary Assay Design Center (Roche Applied Biosciences, Basel, Switzerland). The sequences of the primers were as follows (forward, reverse): COL5A1 (cctggatgaggaggtgtttg, cggtggtccgagacaaag), H19 (ttacttcctccacggagtcg, gagctgggtagcaccatttc), TAGLN (gtccgaacccagacacaagt, acccttgttggccatgtct), RARRES1 (cactactacttggcacagctcac, agtgaatgcgacagggaatta), ALDH1A3 (tggtggctttaaaatgtcagg, tattcggccaaagcgtattc), ID1 (ccagaaccgcaaggtgag, ggtccctgatgtagtcgatga), STAT1 (ttggcacctaacgtgctgt, agttcgtaccactgagacatcct), IFIT1 (agaacggctgcctaatttacag, gctccagactatccttgacctg), MX1 (cggtcctcagcctggtag, tgggggtcccgagatatt), OAS2 (cctgcctttaatgcactgg, atgagccctgcataaacctc), and IFI27 (ccaagcttaagacggtgagg, ccgtggcctagagagtaagaga). The fluorescent TaqMan probes were obtained from the Roche Human Probe Library. The results were analyzed with SDS 2.3 and RQ Manager software (Applied Biosystems), and mRNA expression was determined with the relative quantitation method using beta-actin as an endogenous control. Data were collected from two separate biological experiments, both of which included four replicates of two separate RNA samples.

**Table 1:** Top five differentially expressed genes compared to xenograft.

	Top 5 upregulated genes compared to xenografts	Fold change	Top 5 downregulated genes compared to xenografts	Fold change	Number of differentially expressed genes, up + down, ≥ 2 fold
MG4d	ALDH1A3	24.7	VIM	54.5	797
	C20orf100	15.2	H19	33.8	
	CALB2	12.7	COL5A1	33.7	
	IL8	11.9	MX1	30.1	
	RARRES1	7.8	PMEPA1	24.5	
MG7d	ALDH1A3	29.0	VIM	34.6	473
	RARRES1	28.6	COL5A1	32.4	
	C20orf100	22.8	H19	29.0	
	CALB2	16.2	TAGLN	20.1	
	TOX2	11.0	PMEPA1	19.5	
PH4d	RARRES1	62.9	VIM	63.3	760
	S100P	33.4	TGFBI	26.9	
	ID1	25.3	COL5A1	25.9	
	ALDH1A3	15.5	TAGLN	16.2	
	LXN	12.6	H19	15.4	
PH7d	RARRES1	75.6	VIM	63.1	952
	LCN2	38.5	TGFBI	33.7	
	S100P	32.6	COL5A1	24.7	
	ID1	26.2	H19	20.2	
	LXN	13.9	TAGLN	19.2	
2D	CALB2	23.6	VIM	64.9	2428
	ID1	17.1	H19	27.9	
	ALDH1A3	15.9	COL5A1	26.6	
	LOC100134265	15.5	APOE	13.6	
	RARRES1	13.1	IFIT1	13.5	

Top five up- and downregulated genes and their fold changes in relation to xenograft expression values are shown. Number of up- and downregulated genes is largest in the 2D model.

**Table 2:** Top five differentially expressed genes compared to 2D.

	Top 5 upregulated genes compared to 2D	Fold change	Top 5 downregulated genes compared to 2D	Fold change	Number of differentially expressed genes, up + down, ≥ 2 fold
MG4d	IL8	14.5	CLIC3	6.6	1842
	NCOA7	7.4	LOC100008589	5.6	
	HSP90AA1	5.9	CLDN7	5.5	
	NUFIP2	5.8	ITGB4	5.4	
	CSE1L	5.8	WNT5B	5.2	
MG7d	IL8	9.6	LOC100008589	6.0	2104
	RIOK3	7.1	ID1	5.9	
	TTC3	6.9	GPRC5A	5.8	
	FOSB	6.7	NDUFB9	5.7	
	EIF3E	6.5	SFN	5.0	
PH4d	MX1	25.6	KISS1	13.8	755
	IFIT1	24.6	AXL	12.7	
	OAS2	22.7	WNT5B	10.8	
	IFI27	15.2	MARCH4	10.6	
	STAT1	13.0	FJX1	6.5	
PH7d	HLA-B	35.2	AXL	16.6	851
	OAS2	28.8	KISS1	16.2	
	IFI27	27.8	MT1A	11.2	
	IFIT1	24.8	MARCH4	10.8	
	MX1	24.3	MT2A	10.3	
Xenograft	VIM	64.9	CALB2	23.6	2428
	H19	27.9	ID1	17.1	
	COL5A1	26.6	ALDH1A3	15.9	
	APOE	13.6	LOC100134265	15.5	
	IFIT1	13.5	RARRES1	13.1	

Top five up and downregulated genes and their fold changes in relation to 2D expression values. Number of up- and downregulated genes is larger in the xenograft and Matrigel models than in the polyHEMA model.

### Interferon activity luciferase assays

For the interferon luciferase assays, JIMT1 cells (6000 cells/well) were plated to 96 well plates and grown for 3 days in 2D (in 100 µl), Matrigel (50 µl of Matrigel mix + 50 µl of cell mix), or polyHEMA (in 100 µl) before transfection. On day 3, the cells were transfected with 20 µl of transfection mix containing 0.3 µl of Lipofectamine 2000 (Life Technologies) and 100 ng of reporter plasmid, positive control plasmid or negative control plasmid/well (Cignal ISRE Reporter Dual-Luciferase Assay kit, Qiagen) according to the manufacturer’s instructions. On day 4, STAT1/STAT2-responsive luciferase element activity and the internal control Renilla activity were measured according to the manufacturer’s instructions using 90 µl of Dual-Glo reagent and 90 µl of Stop & Glo reagent. Luminescence was read with the EnVision Multilabel Plate Reader (PerkinElmer).

### Ethics statement

The animal study was approved by the Laboratory Animal Board of Finland (approval number 2008-06166). The study was conducted at the Turku Center for Disease Modeling. Ethical guidelines were strictly followed during the animal work.

## Results

### Setup for high-throughput 3D drug screening

**Figure 1:**
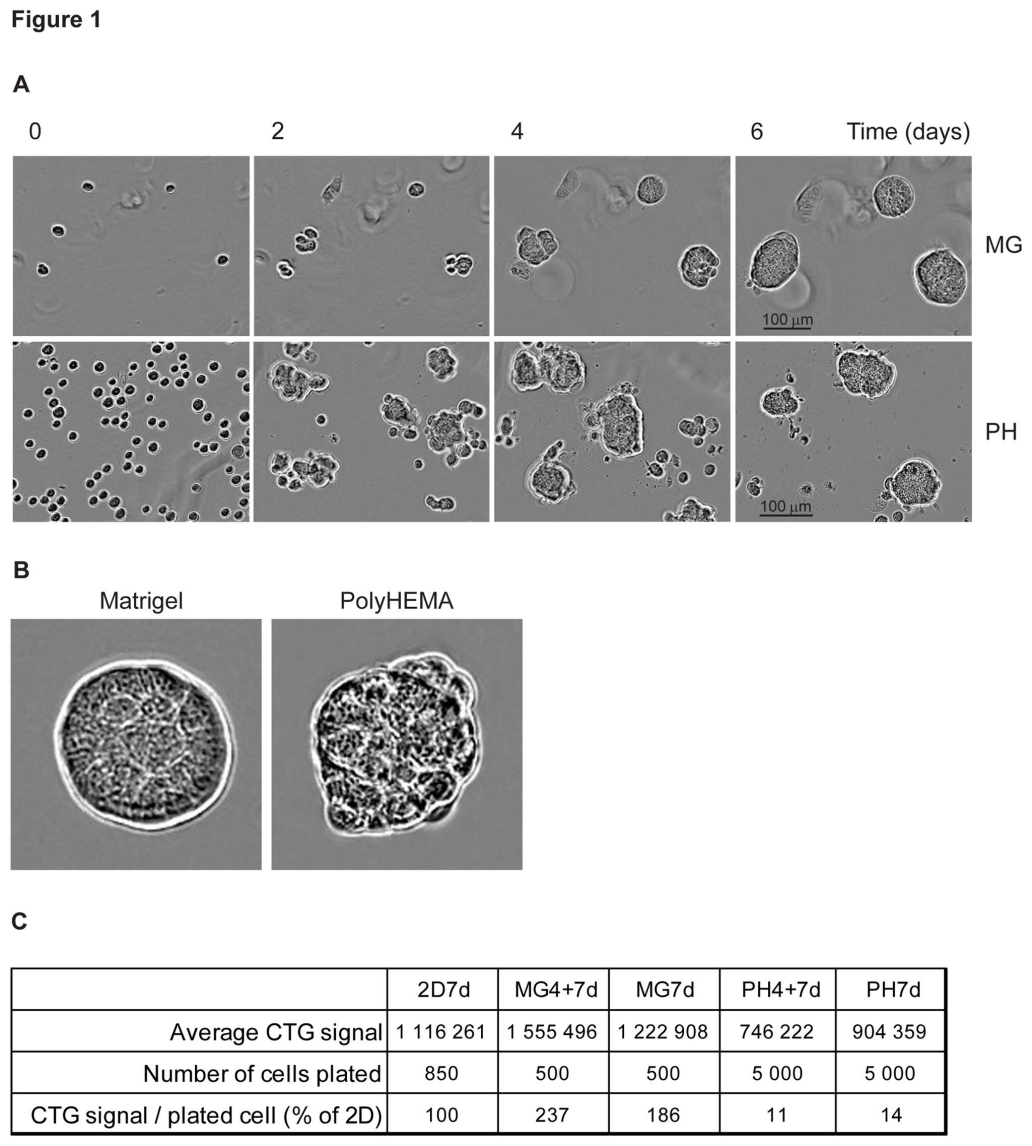
JIMT1 cells form mass-shaped structures in 3D. **A**. Time series of JIMT1 cells grown as either a Matrigel (MG) or polyHEMA (PH) culture. **B**. Zoom-in representative images of mass-shaped JIMT1 structures in Matrigel and polyHEMA. **C**. Growth rate comparison of 2D, Matrigel, and polyHEMA cultures. The growth rate was calculated by dividing the CTG (CellTiter-Glo) read by the starting cell number and expressed as % of 2D. Pictures were taken with IncuCyte.

Transitioning from traditional 2D cultures to 3D cultures has been hampered by the lack of high-throughput assays available for cells grown in 3D and by the high cost due to the increased manual labor involved. We were thus interested in developing a robust and reproducible high-throughput 384-well plate 3D cell culture drug screening assay that could serve as a model for other cell lines, tumor types, and co-cultures and that utilizes automated liquid handling for drug pipetting, cell, and matrix addition. We set up four 3D cell culture model systems and compared drug responses in these to the responses of cells grown in traditional 2D cell culture. The tested 3D culture conditions were: polyHEMA-coated wells for anchorage-independent cell growth as a 7 day culture (PH7d), or as a 4 + 7 day culture (PH4+7d) where the cells were first allowed to form 3D structures for 4 days before addition of drugs, and Matrigel cultures also for 7 days (MG7d), and 4 + 7 days (MG4+7d) (Figure 1A). A 7 day 2D culture was used as a comparison point (2D7d).

The JIMT1 cells grew quite differently in these culture conditions. The cells grew 1.86-fold faster in Matrigel (MG7d) than in 2D cultures (2D7d), and 7.2-fold slower in polyHEMA (PH7d) than in 2D cultures. Thus, the growth rate difference between polyHEMA and Matrigel was 13.52-fold measured with the CellTiter-Glo cell viability endpoint reads divided by the cell number at plating (Figure 1C). In both 3D culture models, the JIMT1 cells formed mass-shaped spheroids [2] although they were more uniformly shaped and sized in Matrigel (Figure 1B). PolyHEMA cultures contained more single cells as well as larger structures. The cell growth rate in the different models was further analyzed with cell viability growth curves, and induction of apoptosis was studied by caspase-3/-7 activity assays, which indicated increased apoptosis in the polyHEMA cultures (File S2). In addition, a more extensive panel of representative images in the presence and absence of a drug is provided in File S3.

### Drug screening revealed marked differences between the cell culture models

To compare drug responses between the different 3D cell culture conditions, JIMT1 breast cancer cells were screened with two different drug libraries: a MicroSource cancer compound library with 80 compounds in four dilutions, each in one replicate, and a custom 22 compound library mainly consisting of HER2+ cancer-targeted drugs in seven dilutions, each in two intra-plate replicates (File S1). All screens were carried out in three biological replicates.

**Figure 2:**
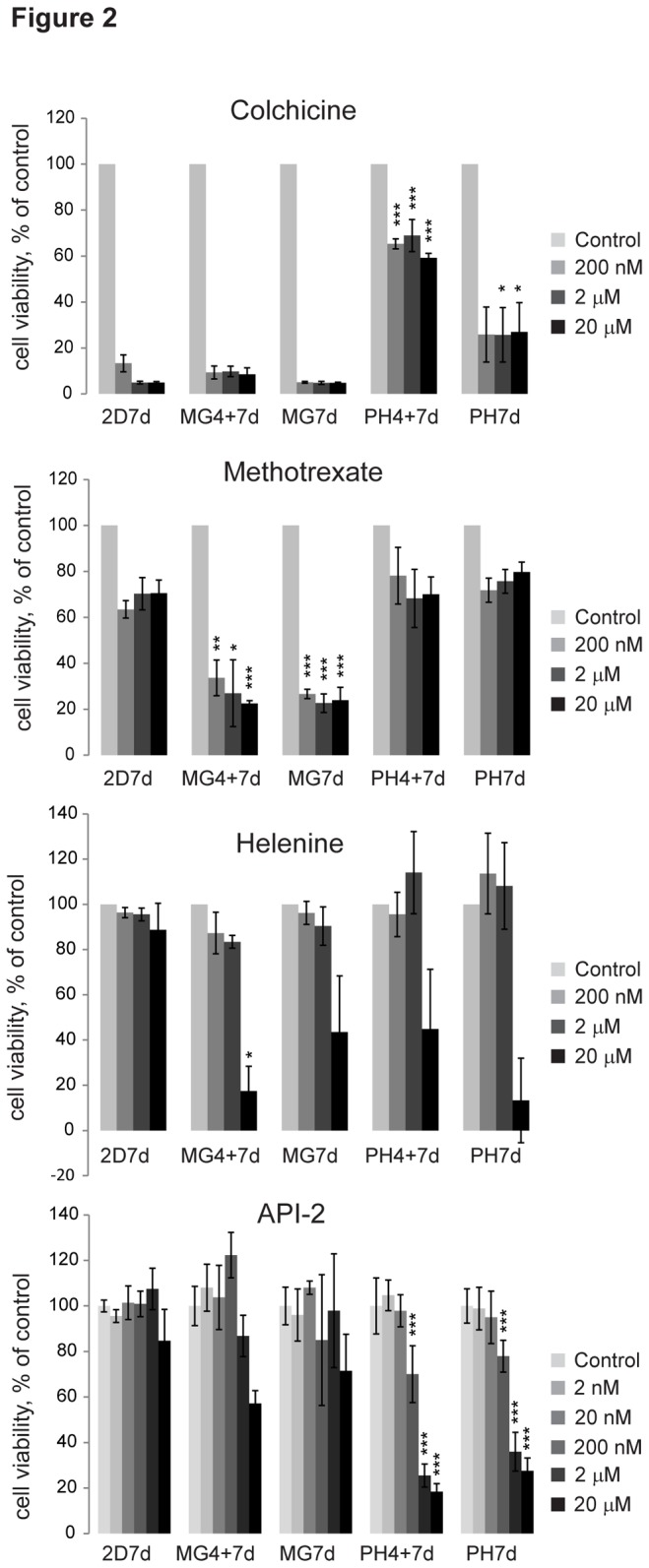
Drug screening shows differences between the culture models and responses to individual drugs vary greatly. Cells cultured in 2D, Matrigel (MG4+7d, MG7d), or polyHEMA (PH4+7d, PH7d) were treated with colchicine, methotrexate, helenine or API-2 with the indicated concentrations for 7 days. The cells were treated either directly up on plating for 7 days (2D7d, MG7d, PH7d) or after 4 day pre-growth at 3D (MG4+7d, PH4+7d). CellTiter-Glo (CTG) was used as a cell viability measure; data are an average of three biological replicates. Error bars are STDEV, * indicate statistically (t-test) significant changes compared to the corresponding 2D7d concentration, * < 0.05, ** < 0.01, *** < 0.001.

Interestingly, the cells responded differently to several drugs in the different cell culture models. Colchicine, a microtubule polymerization inhibitor traditionally used for treating gout and currently being investigated for use as an anticancer drug, was highly effective in inhibiting cell viability in the 2D and Matrigel models, but the drug showed only moderate effects in the polyHEMA models (Figure 2).

Methotrexate is an antimetabolite and antifolate drug developed for use in cancer treatment and for autoimmune diseases. Methotrexate showed clear growth inhibition in the Matrigel models but only modest growth inhibition in the 2D and polyHEMA models (Figure 2). This drug acts on most rapidly dividing cells and could thus be expected to be most efficient in the Matrigel models. However, the effect in the polyHEMA and 2D models was very similar although the growth rate was 7-fold higher in 2D models (Figure 1C).

Helenine (Alantolactone), an antimycobacterial agent, which has been hypothesized to inhibit cell proliferation by inducing activin/SMAD3 signaling [27] inhibited growth in all 3D models but showed little effect in the 2D models (Figure 2).

In addition, the Akt pathway inhibitor API-2 was clearly most effective in both polyHEMA models, showed only a minor effect in Matrigel, and was ineffective in the 2D models (Figure 2). The API-2 effect was unique among the Akt/PI3k inhibitors used in the study.

As shown in Figure 2, individual drug responses can differ greatly among the five different cell culture models and can show sensitivity advances to any of the models. Therefore, generalizations of cell culture model-specific drug sensitivities cannot be made based on effects seen with a single drug. This has been the case in many previous studies comparing 3D cell cultures to 2D cultures.

### Cells grown in Matrigel are most sensitive to drugs

**Figure 3:**
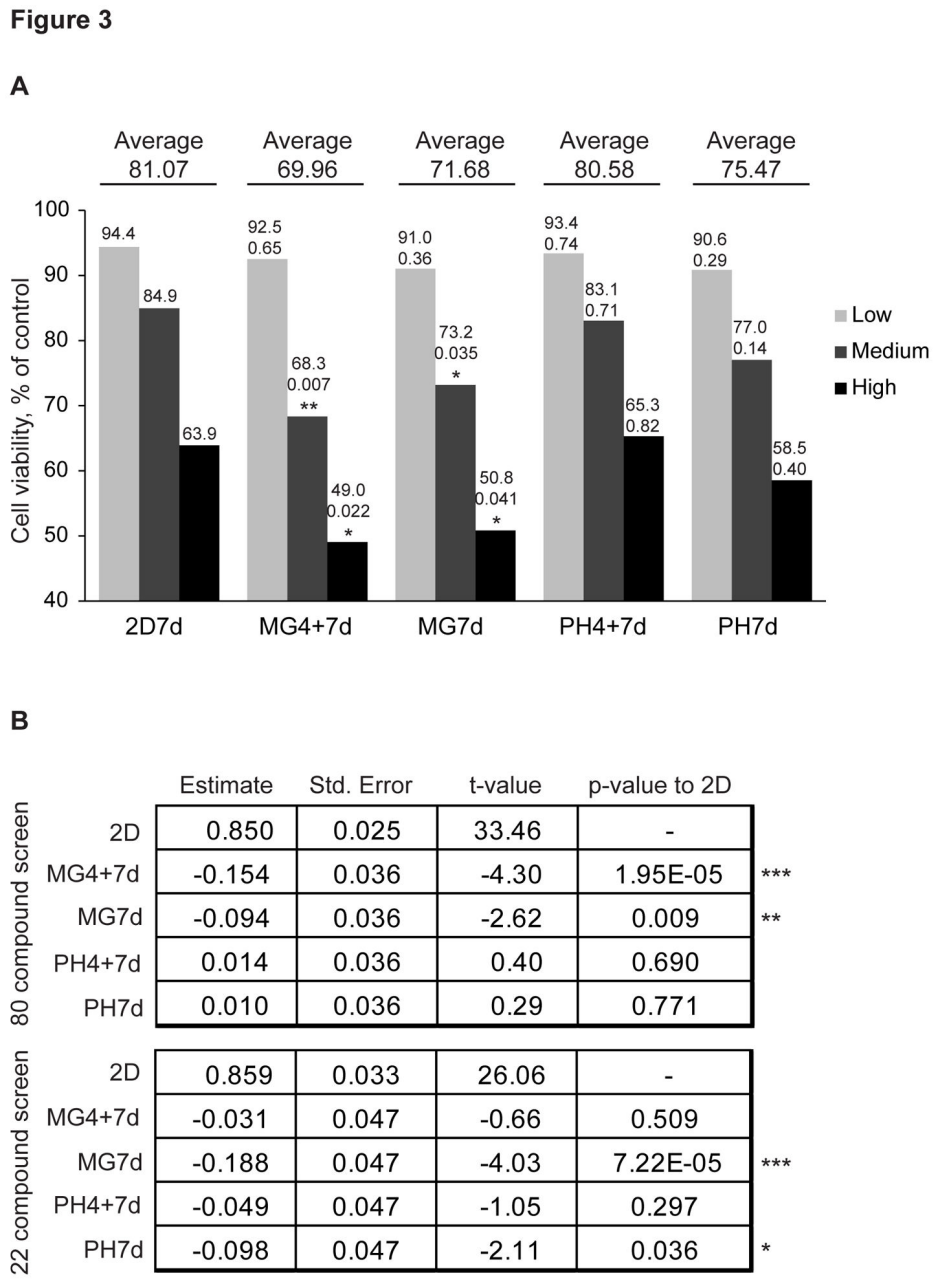
JIMT1 cells are more sensitive to drugs in Matrigel. **A**. Average drug response of the JIMT1 cells in different cell culture models. The average responses of the 63 drugs are shown individually for the three highest concentrations (high (0.34-20 µM), medium (0.034-2 µM), and low (0.0034-0.2 µM) and across the concentrations. Response, p-value, and significance (** < 0.01, * < 0.05) compared to 2D are shown above the bars and average across the concentrations is shown above the line. Drug annotations and concentrations used for each drug are shown in File S1. **B**. Multiple linear regression analysis of individual drug screens comparing general drug effects to 2D. T-value shows the response difference to 2D (33.46 or 26.06). ***< 0.001, **<0.01, *<0.05.

As our goal was to compare drug effects between the different cell culture models in general, we combined the results from both drug screens. Out of 102 drugs, 63 inhibited cell viability more than 30% in at least one of the models and were selected for further analysis. The average of the 63 drugs’ three highest concentrations showed that cells in the 2D model had the smallest response to drugs (81.07% of the control value). Cells were significantly more responsive to the drugs in both Matrigel models than in the 2D model with the two highest concentrations (Figure 3A). Despite the slower growth rate, cells were slightly more responsive to these drugs in the polyHEMA models than in the 2D model, but the findings were not statistically significant.

To verify these findings, a separate bioinformatic analysis was conducted using the raw screening data. In this analysis, the two drug screens were analyzed separately, and all the drugs were included. Concentration-wise multiple linear regression analysis was performed for the MicroSource cancer compound library screen with 80 compounds. Overall, for every unit increase in treatment response in the 2D cell culture model, there was a further increase in response in MG7d (estimate -0.154, p-value < 0.001) and in MG4+7d (estimate -0.094, p-value < 0.01). Similarly, in the smaller screen containing 22 drugs, the analysis showed that cells in MG7d were significantly more responsive to drugs than in the 2D model (estimate -0.188, p-value < 0.001) (Figure 3B).

These two independent analyses indicate that JIMT1 cells are in general more responsive to drugs when grown in Matrigel than in traditional 2D cultures. However, as shown in Figure 2, there are some exceptions; therefore, the effects of individual drugs should not be generalized. The result could also explain some of the diversity in the literature reporting sensitizing and desensitizing effects in 3D culture models.

### Gene expression patterns of cells grown in Matrigel cultures are closest to xenografts

Three-dimensional models are considered to resemble *in vivo* conditions more than 2D monolayer cultures [28]. To verify this, we compared the gene expression profiles of all our cell culture models to the JIMT1 xenograft expression profiles. JIMT1 cells were injected into the mammary fat pads of mice, and the tumors were collected 43 days later. To understand the different models better, we analyzed the gene expression patterns of 4 day and 7 day old cultures. It takes 4 days for the cells to form 3D structures. Therefore, we postulated that this could be a critical time-point to see expression changes compared to the 2D cultures. Genome-wide gene expression levels of tumor samples and culture model samples (xenograft, 2D7d, MG4d, MG7d, PH4d, and PH7d) were obtained with Illumina HumanHT-12 v4 Expression BeadChip. Averages of duplicate samples were used for data analysis.

**Figure 4:**
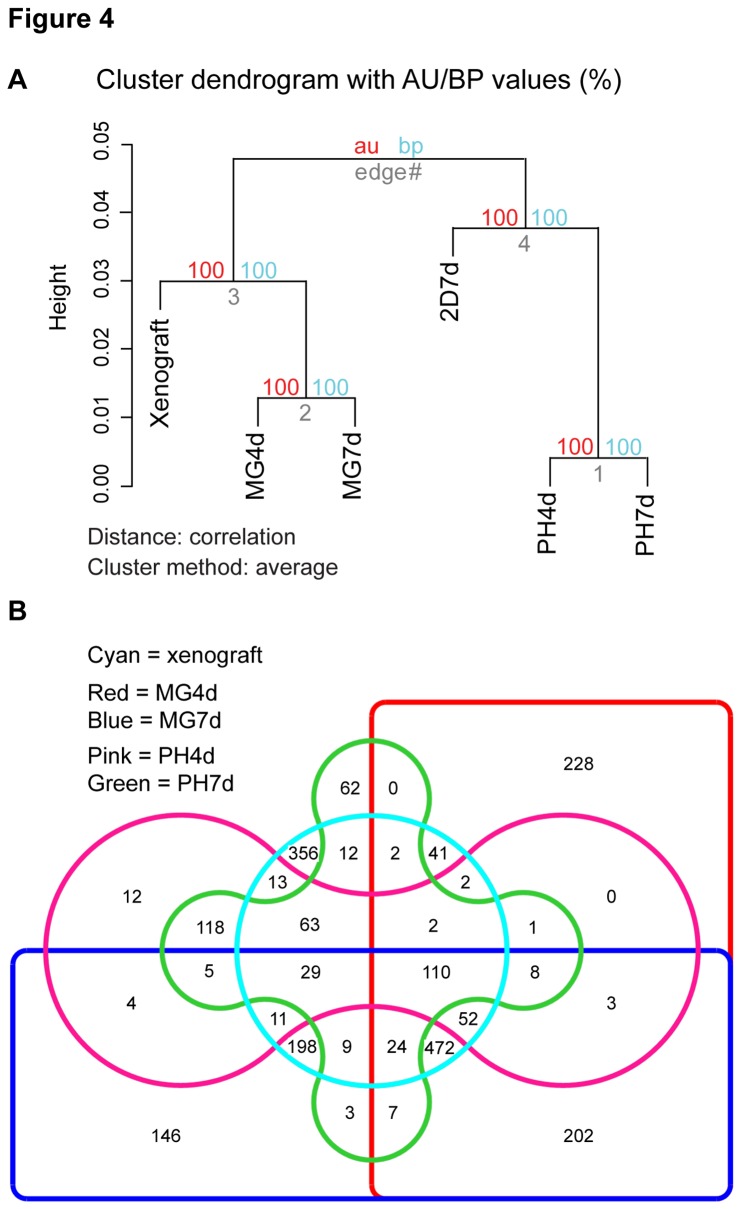
Gene expression of JIMT1 cells grown in Matrigel is closest to xenografts. **A**. Dendrogram showing hierarchical clustering of genome-wide gene expression data. au = approximately unbiased p-value (%) in red, bp = bootstrap probability value (%) in blue, edge = cluster number in gray. Data shown are the average of two biological RNA replicates. Correlation of the replicates is shown in File S6 B. Venn diagram of ≥2-fold upregulated genes compared to 2D7d shows the number of shared genes in every combination of culture models. A list of common genes for each category is shown in File S4.

Unsupervised hierarchical clustering of the data groups Matrigel cultures (4 day and 7 day samples) together with the xenografts, and polyHEMA cultures cluster together with 2D cultures in a separate branch. The au (approximately unbiased) and bp (bootstrap probability) p-values for these clusters were 100% for each relation indicating statistically highly significant differences (Figure 4A). The data indicate that JIMT1 Matrigel 3D cultures resemble xenograft cultures more closely than 2D or polyHEMA cultures. However, this cannot be generalized. Similar samples (xenograft, 2D7d, MG4d, MG7d, PH4d, and PH7d) were prepared from the HER2-negative, ER-positive breast cancer cell line MCF7. The overall gene expression changes between the models were minimal, and no co-clustering of Matrigel samples with xenografts was observed (GEO accession number GSE47583).

The VENNTURE [22] Venn diagram of the differentially expressed genes (≥2-fold upregulated) compared to the 2D culture shows that Matrigel cultures share 472 commonly upregulated genes with xenografts whereas polyHEMA cultures share only 63 commonly upregulated genes with xenografts (Figure 4B). In addition, the data indicate that cells in Matrigel continue to differentiate toward a more xenograft-like signature as the MG7d signature shares 1416 up/downregulated genes with the xenografts when compared to the 2D cultures whereas the MG4d model shares 1004 genes with the xenografts. This was not the case in the polyHEMA cultures as both PH-profiles shared 515 up/downregulated genes with the xenografts compared to the 2D cultures (Figure 4B, File S4).

In addition, there were 54 commonly downregulated genes in the 3D cultures and in the xenografts when compared to 2D. Ingenuity pathway analysis (IPA) showed that these genes are involved in ER pathway activation and hepatocyte growth factor, transforming growth factor, and synovial apoptosis inhibitor 1 pathway inhibition, indicating that there are significant changes in hormone and growth factor signaling when cells are cultured in 2D (files S4 and S5).

Correlation analysis of the genome-wide data shows high overall correlation of the samples; xenografts had the weakest correlation with 2D7d (average 0.923), medium correlation with the polyHEMA models (average 0.951), and the highest correlation with the Matrigel models (average 0.966) (File S6). A closer look at the data revealed that the number of differentially expressed genes (more than 2-fold, up and down) between the xenografts and the 2D7d was 2428, the xenografts versus the PH7d was 952, and the xenograft versus the MG7d was 473 genes (Table 1), indicating that xenografts had a closer relationship with the Matrigel cultures than to the others tested.

**Figure 5:**
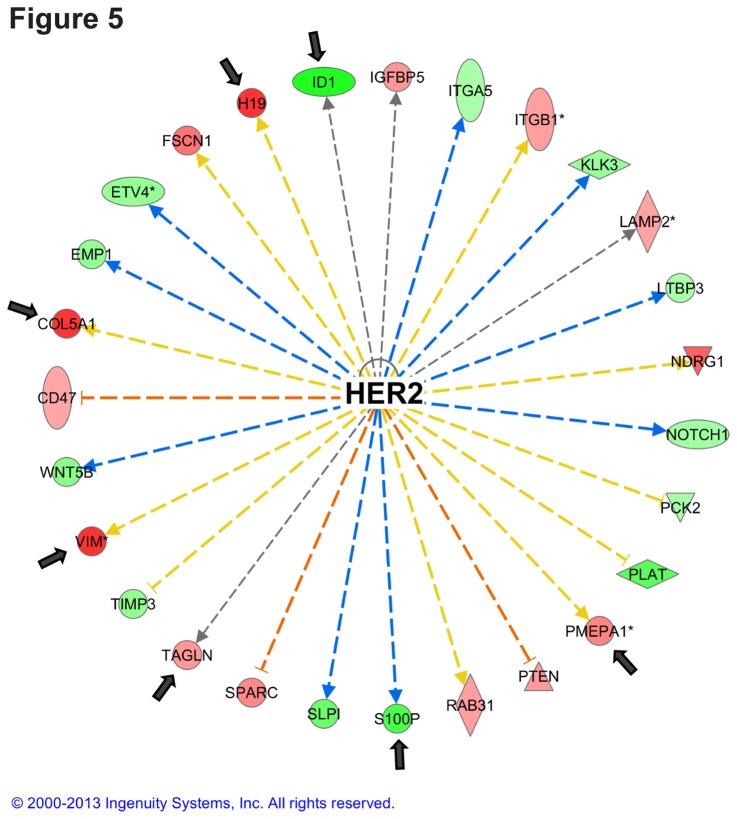
HER2 signaling is altered in xenograft. The most significantly altered upstream regulator in xenograft compared to 2D according to IPA; the HER2 pathway with arrows pointing to the top five changing genes shown in Table 1. Genes in red were upregulated in xenografts, and genes in green were downregulated. The 200 most upregulated and the 200 most downregulated genes in the xenografts compared to the 2D cultures were subjected to IPA analysis using the log-fold differences in expression as comparison values.

The most differentially upregulated gene in xenografts compared to 2D, Matrigel, or polyHEMA was vimentin (64.9-, 34.6- to 54.5-, 63.1- to 63.3-fold expression difference, respectively), which could indicate an epithelial-to-mesenchymal transition (EMT) [29]. From the top five downregulated genes when compared to xenografts, TAGLN and COL5A1 have also been indicated in EMT [30,31]. The top five downregulated entries from all the comparisons to xenografts contained only nine unique genes (APOE, COL5A1, H19, IFIT1, MX1, PMEPA1, TAGLN, TGFBI, and VIM) (Table 1). Interestingly, most of these genes (COL5A1, H19 PMEPA1, TAGLN, and VIM) are activated by HER2 signaling according to IPA. Furthermore, S100P and ID1, which were in the top five upregulated in polyHEMA and 2D models compared to the xenografts are downregulated by HER2 signaling (Figure 5). According to IPA, HER2 is the most significantly changing upstream regulator in xenografts when compared to the 2D cultures (p-value 1.15E-13, activation z-score -1.05, File S5) indicating that although HER2 signaling is only slightly downregulated in xenografts, it is significantly altered when compared to traditional 2D cell culture models. The HER2 signaling pathway was also significantly altered in the polyHEMA and Matrigel 3D cultures compared to the 2D cultures, indicating that HER2 signaling sensitively changes in response to differences in the extracellular environment. This is essential to keep in mind when studying HER2 signaling. IPA gene expression analysis for changes in upstream signaling was carried out for three additional cell lines. Statistically significant changes in HER2 signaling pathway activity between the xenograft and 2D cultures were also observed in MCF7 HER2-negative, ER-positive breast cancer cell line and in the MDA-MB-231 and Hs578T triple negative breast cancer cell lines (GEO Series accession number GSE47583 for MCF7 and GSE36953 for MDA-MB-231 and Hs578T cell lines).

**Figure 6:**
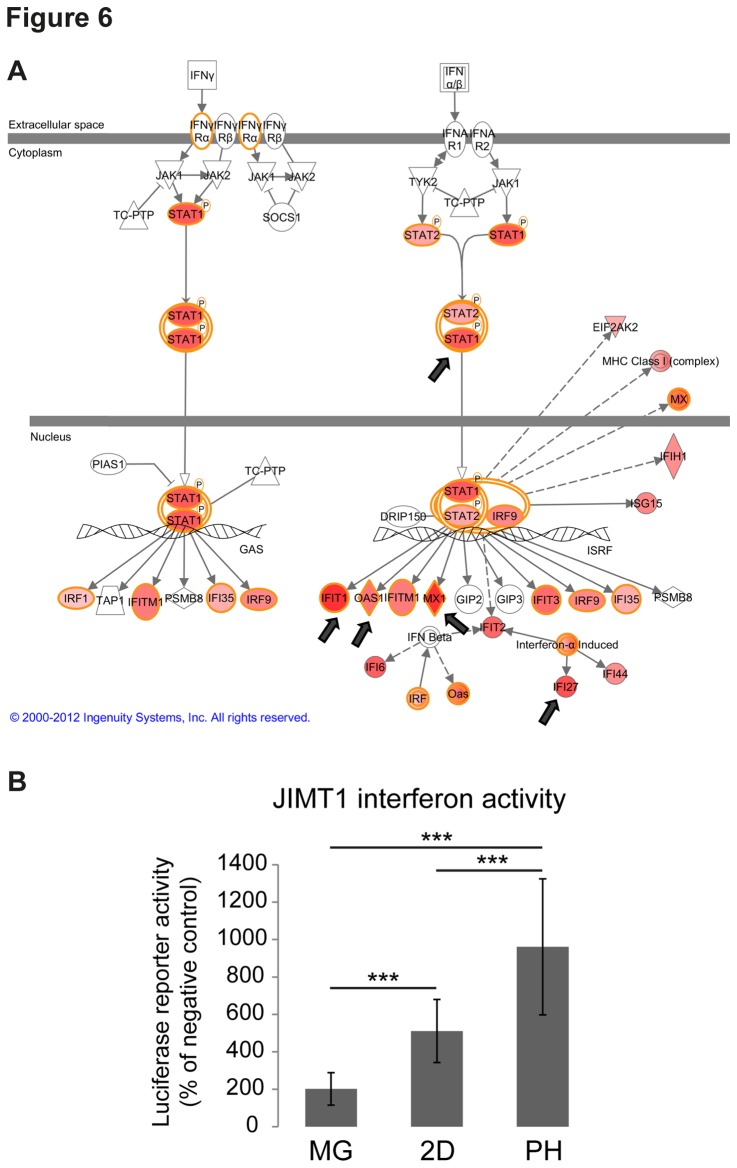
Interferon pathway is activated in polyHEMA cultures. **A**. The canonical pathway that changed the most in PH4d and in PH7d compared to 2D according to IPA; the interferon pathway with arrows pointing to the top five changing genes in PH4d is shown in Table 2. Genes in red were upregulated in PH4d. Two hundred of the most up- and downregulated genes in PH4d/PH7d compared to the 2D cultures were subjected to IPA analysis. **B**. The gene expression results were validated by measuring interferon-alpha and beta activity from JIMT1 cells by transfecting them with a Cignal ISRE Reporter dual-luciferase assay kit (Qiagen). The kit measures induction of the STAT1 and STAT2 components of the JAK/STAT-signal transduction pathways as a readout for interferon activity. The cells were grown in 2D, MG, or PH for 3 days before reporter plasmids and transfection reagents were added for 24 hours. *** p-value < 0.001.

Interestingly, the most changing pathways in the xenografts when compared to the 2D cultures were the interferon signaling and PI3K/Akt pathways (File S5). In addition, the polyHEMA models had a very clear interferon response with many of the interferon pathway components highly upregulated compared to the 2D cultures. Although interferon signaling was also high in xenografts, it was still markedly higher in polyHEMA (Figure 6, Table 2, File S5). Dual luciferase interferon activity assays were conducted to validate these findings. The assays showed significantly increased interferon pathway activation in the polyHEMA cultures and in the 2D cultures when compared to the Matrigel cultures (Figure 6B). All of the top five upregulated genes in PH4d compared to the 2D cultures are part of the interferon pathway: MX1, IFIT1, OAS2, IFI27, and STAT1 (Figure 6A). In general, all of the 3D and xenograft models were altered from 2D by differential expression of genes involved in cellular movement, cell-to-cell signaling and interactions, and cell death and survival (File S5).

This analysis indicates that the gene expression of cells grown in Matrigel models most closely resembles the gene expression of the xenografts. Therefore, the overall drug response of JIMT1 cells grown in Matrigel cultures is likely to most accurately represent xenograft drug responses. To validate the gene expression array results, the RNA expression levels of 11 genes were determined with TaqMan RT-PCR. The results clearly supported the array data (File S7).

Changes in the gene expression patterns could also explain some of the differences in drug responses observed in Figure 2. We showed that helenine, which has been hypothesized to inhibit cell proliferation by inducing activin/SMAD3 signaling [27], inhibited growth in all 3D models but showed little effect in the 2D model (Figure 2). This could possibly be due to increased SMAD3 signaling dependency in the 3D models as we observed higher p21 (activated by SMAD3) and lower c-myc expression levels (inhibited by SMAD3) in the 3D models compared to the 2D model. The p21 levels were 11% and 162% higher in MG7d and PH7d, respectively, than in the 2D model whereas c-myc expression was 49% and 45% lower in MG7d and PH7d, respectively, than in the 2D model (GEO Series accession number GSE42529 for gene expression results).

In contrast, we observed that the Akt pathway inhibitor API-2 was clearly the most effective in both polyHEMA models (Figure 2). This was unexpected as the gene expression profiling of the polyHEMA model did not indicate activation of the Akt pathway when compared to either the 2D or Matrigel model; instead, PTEN was activated with a Z-score of 2.62 (p-value 2.24E-9) when compared to 2D indicating that the Akt pathway is inhibited (File S5).

## Discussion

The aim of the study was to develop and test various 3D culture models suitable for high-throughput drug screening approaches utilizing automated liquid handling as much as possible to reduce manual labor and human error. Automated liquid handling was easily adaptable for the 7 day models; PH7d required the 384 well plates to be pre-coated with polyHEMA before drug pipetting, and MG7d required the creation of Matrigel gel on the drug-containing plates before the cells were added. Matrigel and the equipment (automated plate-filler cassettes, cleaning buffers, and plates) had to be kept ice-cold for the entire procedure to avoid polymerization of Matrigel to the machines. No Matrigel was added to the culture medium as is often done [32]; this was left out from the protocol to avoid unnecessary stress to the cells caused by the ice-cold medium that would have been needed to add Matrigel. However, no apparent morphological differences were detected in our 3D cultures in Matrigel when compared to images of cell spheres in the literature where additional matrix was used.

The robotic system needed to be adjusted for the 4+7 day treatments so that the drug libraries could be pipetted on top of the growing cultures at day 4. In the 4+7d models, cells had time to form 3D structures before interference with the compound. The hypothesis was that this model would mimic *in vivo* conditions better, as the cells were allowed to form 3D structures before the drug treatments. However, the 4+7d model is more laborious and more expensive to carry out than the 7d model, which is technically more similar to high-throughput screening of 2D cultures. The gene expression analysis indicated that the expression profile of cells grown in Matrigel continues to develop toward a xenograft-like profile as the longer 7 day cultures shared a larger number of differentially expressed genes with xenografts than the shorter 4 day cultures when compared to the cells in the 2D cultures. Interestingly, this was not the case in the polyHEMA cultures. Furthermore, cells grown in the MG4+7d model were most different from the 2D model in the overall drug response analysis, thus supporting our hypothesis although there was only a minor, non-significant, difference from the MG7d model. All the drug screens were carried out for a minimum of 7 days, so the potential impact of gene expression changes at the 4 and 7 day time points to drug responses is hard to judge. Based on the similarities in the drug screening results between the two Matrigel models and ease of use, we believe the Matrigel 7 day protocol (MG7d) is the most practical alternative for JIMT1 high-throughput 3D drug screening. For other cell lines, the most suitable 3D high throughput approach needs to be determined case by case.

We also note that the gene expression comparison of xenografts to cells grown on Matrigel cultures is somewhat biased as the cells were injected in the fat pads of mice together with 25 µl of Matrigel to improve the initiation of tumor growth. However, the tumors were collected 43 days after inoculation, and the amount of Matrigel at the beginning of the experiment was so small that it is unlikely to have affected the tumour gene expression at the end of the experiment.

Our drug screening results show that no general conclusions on drug sensitivities in different culture conditions can be drawn based on effects seen with a single drug. This is in line with the literature; Weigelt et al. have shown that AU565 breast cancer cells grown in Matrigel cultures are more sensitive to the HER2 inhibitory antibody trastuzumab but less sensitive to HER2 dimerization inhibiting antibody pertuzumab when compared to the same cells grown in 2D cultures [10]. Others have also shown that cells cultured in 3D matrices are either sensitized or desensitized to drug treatment, depending on the cell and/or drug type [33,34].

However, analysis of the entire screening data clearly showed with two independent analysis methods that JIMT1 cells are sensitized to drugs when grown in Matrigel cultures. To our knowledge, this is the first time general drug sensitivities between cell culture models have been analyzed in a high-throughput manner. These results support previous discoveries that the extracellular matrix and microenvironment play a significant role in cell responses [2,35,36].

Our results indicate that data from 2D drug responses of JIMT1 cells may not represent the *in vivo* responses very well as they differ significantly from the closest *in vivo* counterpart for JIMT1 cells tested here, the Matrigel cultures. Three-dimensional culture models have also been indicated to better mimic *in vivo* situations by others; EMT-6 mammary tumor cells are highly resistant to alkylating agents *in vivo* and lose their resistance in traditional 2D monolayers as opposed to 3D cultures [37]. In addition, the cytotoxic effect of Adriamycin in the spheroid culture model more closely parallels the *in vivo* effect than the monolayer cultures [16]. However, this might not always be the case. Our examination of the gene expression patterns of the MCF7 2D, Matrigel, polyHEMA, and xenograft cultures showed that the differences between the culture models were much smaller in the MCF7 cells than in the JIMT1 cells indicating that the differences between drug responses might be much smaller.

As we observed a marked difference in growth rates between the different culture models tested, one could argue that the detected differences in drug sensitivities are due to varying growth rates. However, Heiser et al. studied the effects of 77 compounds in 50 breast cancer cell lines and concluded that the drug responses were not strongly influenced by the growth rates of breast cancer cells [38]. On the contrary, they observed that half of the luminal breast cancer subtype-specific compounds were most effective in the most slowly growing cells. Similarly, comparison of drug responses of fast growing leukemia cells (L1210) and ten human tumor cell lines (5 melanomas, 4 colon carcinomas and 1 small cell lung carcinoma) did not show increased resistance in the slower growing tumor cell lines [39]. Furthermore, if the drug responses were directly comparable to growth rates, cells grown in polyHEMA cultures should clearly have been the least sensitive to drugs in our assays, and that was not the case; cells grown in 2D cultures were.

It is possible that the growth rate measured by comparing the starting cell number to the endpoint cell viability is suboptimal for polyHEMA cultures as the cells were plated in high numbers and formed tight and large mass-shaped structures. Nutrients thus might not reach the cells within the center, and the cells might stay in a quiescent, or even necrotic, state not having ATP-levels comparable with the proliferating outer layer [5,40]. Mouse mammary tumor cell line spheroids in collagen have been shown to contain more senescent cells and increased necrosis compared to monolayer cultures [41]. We found evidence of increased cell death in the polyHEMA cultures as we observed higher caspase-3/-7 activity in the polyHEMA cultures than in the 2D or Matrigel cultures.

Differences in cell number might affect drug responses. For example, colon cancer cells become more resistant to drugs in confluence dependent manner [42-44]. This could affect interpretation of the polyHEMA drug screening data as these cultures were seeded in a much higher density than 2D and Matrigel cultures. In addition, serum concentration has been shown to affect chemosensitivity; ovarian cancer cell line OVCA433 was clearly more sensitive to 4 cytotoxic drugs (4-HC, Cisplatin, Paclitaxel, and Topotecan) when cultured in 10% serum instead of serum free complete assay medium [45]. In our study, the cells cultured in Matrigel were grown in 6% serum whereas 2D and polyHEMA cultures had 10% serum. Based on the Fernando et al. study the difference in serum concentration would increase the drug sensitivity of 2D and polyHEMA cultures in respect to Matrigel cultures which was not the case. Matrigel, although growth factor reduced, does include similar growth factors and nutrients as serum, and could thus even out the difference in serum concentration.

As previous studies using single agents have suggested, and which was confirmed here in JIMT1 breast cancer cells using 63 compounds, cells grown in 3D Matrigel cultures show an increased responsiveness to anticancer compounds [2,9-11,46]. The reason is still unclear, but one possible explanation is tumor heterogeneity; 2D and polyHEMA cultures might give growth advantage to a more drug resistant cell population than Matrigel cultures [47]. Another explanation is that cells in 3D cultures use paracrine mechanisms similar to *in vivo* tumors and that these mechanisms do not exist in 2D monolayers [48]. This is supported by data from our gene expression arrays; the cytokine IL8 was the most upregulated gene in both Matrigel (4 day and 7 day) samples. Interestingly, IL8 has been linked to a specific “HER2/HER3 signature” supporting our findings on changes in HER2 pathway activities in 3D cultures [49]. Notably, examining public datasets revealed high levels of IL8 transcripts in HER2-enriched as well as basal-like primary breast tumors, two subtypes characterized by a particularly poor prognosis. Moreover, IL8 expression correlated with high tumor grade and ER-negative status.

Naturally, these observations made in JIMT1 breast cancer cells cannot be directly applied to other cell lines, and the preferred growth conditions have to be determined cell line by cell line. Also, changes in drug sensitivity between the culture models should be tested using additional cell lines to confirm our findings. Optimally, the observed differences in drug sensitivities should in parallel be tested in xenografts. However, this study indicates that JIMT1 cells are most sensitive to drugs and most equal to *in vivo* conditions when grown in Matrigel as 3D cultures. Interestingly, significant differences in growth factor and HER2 signaling were observed between the xenograft and 2D cultures, pointing to the limitations of the traditional 2D cell culture method.

## Supporting Information

File S1 **Drug screen plate annotations and screening results.** Sheet 1+3: Annotations of 22 drugs and MicroSource 80 compound libraries used in the screens. Sheet 2+4: Loess-log normalized drug screening results.
(XLSX)

File S2 **Characterization of the models.** **A**. Growth curves of JIMT1 cells grown as 2D, Matrigel, or polyHEMA cultures. Median cell viability values (CellTiter-Glo) and STDEV from 16 wells are shown. B. Caspase-3 and -7 activity (Caspase-Glo 3/7 assay, Promega) from 2D, Matrigel (MG), or polyHEMA (PH) cultures measured at 9 or 13 days of culture. The averages of six wells with STDEV are shown, p-value < 0.001 for all changes.
(EPS)

File S3 **Representative image of the models.** Representative images of JIMT1 cells in 2D (2D7d), Matrigel (MG4+7d), or polyHEMA (PH4+7d) cultures grown up to 11 days in the presence or absence of 5 µM API-2. Images are taken from 384 well plates using IncuCyte (Essen Bioscience).
(TIF)

File S4 **Commonly up- or downregulated genes in each model.** Sheet 1: VENNTURE image of downregulated genes compared to 2D expression. Sheet 2: VENTURE gene list of upregulated genes compared to 2D. Sheet 3: VENNTURE gene list of downregulated genes compared to 2D. Sheet 4: VENNTURE place code.
(XLSX)

File S5 **IPA pathway analysis of gene expression changes.** Sheet 1: Commonly downregulated genes in all of the 3D models and xenografts compared to the 2D cultures Sheet 2: HER2 is the most significantly changing up-stream regulator in xenografts when compared to 2D. Sheet 3: The canonical pathways that changed the most in xenografts compared to 2D cultures: the interferon pathway and the PI3K pathway. Interferon signaling is upregulated in polyHEMA cultures compared to xenografts. Sheet 4: Top five changing molecular and cellular functions. Sheet 5: IPA top changing upstream regulators in PH7d versus 2D7d. PTEN is significantly activated in polyHEMA.
(XLSX)

File S6 **Correlation analysis of gene expression profiles.** Sheet 1: Genome-wide Pearson correlation is shown separately for the two biological repeats.
(XLSX)

File S7 **TaqMan RT-PCR validation of gene expression results.** Gene expression of 11 genes was validated with TaqMan RT-PCR. A. Genes based on gene expression analysis were downregulated in the cell culture compared to xenografts. B. Genes based on gene expression analysis were upregulated in the cell culture compared to xenografts. C. Genes based on gene expression analysis were upregulated in polyHEMA compared to xenografts. D. Genes based on gene expression analysis were upregulated in polyHEMA compared to 2D cultures. The data shown are an average of 16 replicates (two biological replicates each including four repeats of two RNA replicates).
(EPS)
