# Supplementary figures and images for: High-Throughput 3D Screening Reveals Differences in Drug Sensitivities between Culture Models of JIMT1 Breast Cancer Cells
Source: PLoS One. 2013 Oct 23;8(10):e77232. doi: 10.1371/journal.pone.0077232 (PMC3806867; doi:10.1371/journal.pone.0077232)

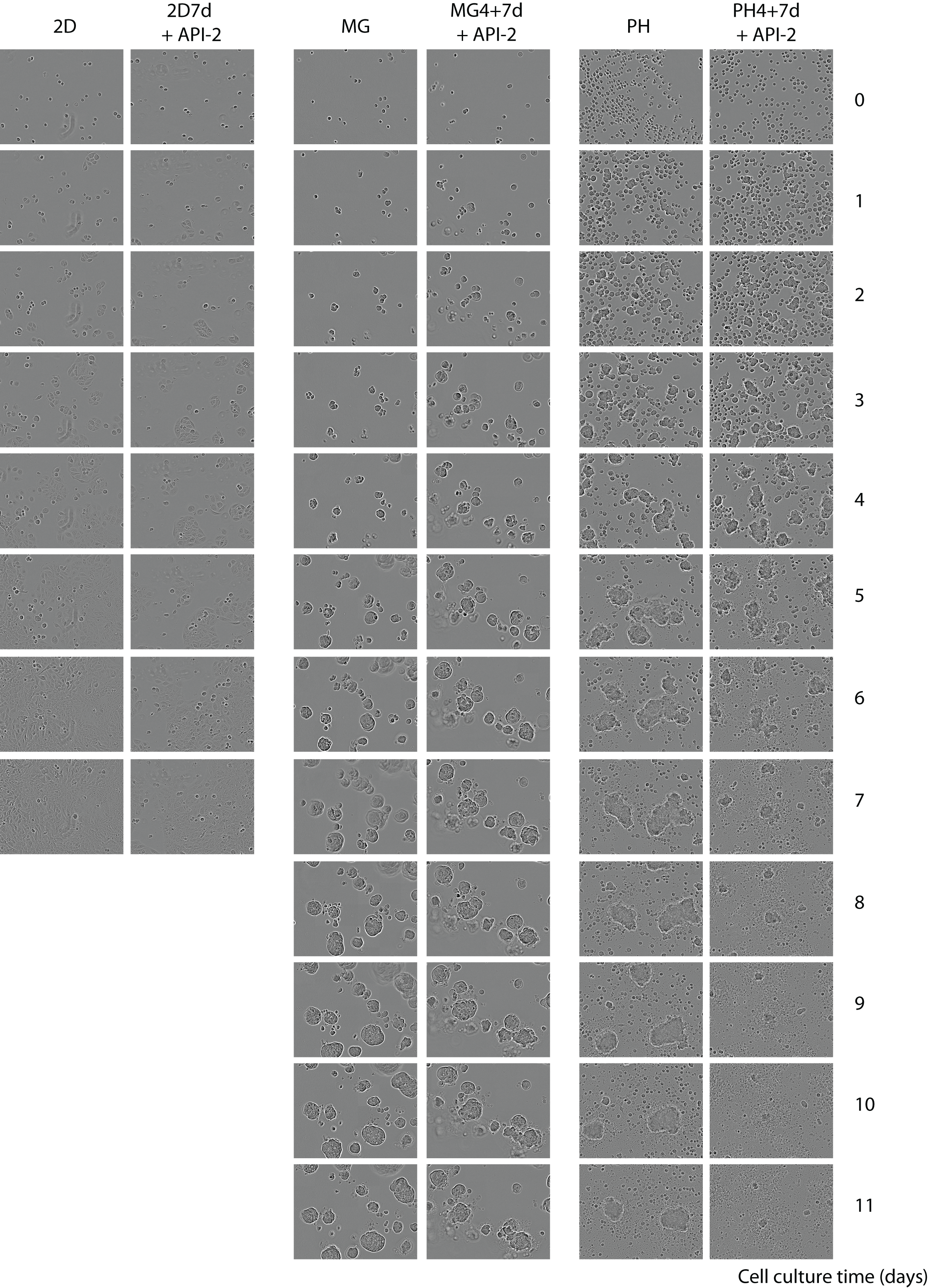

Supplement: File S3 — Representative image of the models. Representative images of JIMT1 cells in 2D (2D7d), Matrigel (MG4+7d), or polyHEMA (PH4+7d) cultures grown up to 11 days in the presence or absence of 5 µM API-2. Images are taken from 384 well plates using IncuCyte (Essen Bioscience). (TIF) [file pone.0077232.s003.tif]
